# Local eukaryotic and bacterial stream community assembly is shaped by regional land use effects

**DOI:** 10.1038/s43705-023-00272-2

**Published:** 2023-06-26

**Authors:** Benjamin Weigel, Caio Graco-Roza, Jenni Hultman, Virpi Pajunen, Anette Teittinen, Maria Kuzmina, Evgeny V. Zakharov, Janne Soininen, Otso Ovaskainen

**Affiliations:** 1 Research Centre for Ecological Change, Organismal and Evolutionary Biology Research Programme, Faculty of Biological and Environmental Sciences, University of Helsinki, P.O. 65, FI-00014 Helsinki, Finland; 2 INRAE, EABX, 50 avenue de Verdun, 33612 Cestas, France; 3 Laboratory of Ecology and Physiology of Phytoplankton, Department of Plant Biology, State University of Rio de Janeiro, Rua São Francisco Xavier 524, PHLC, Sala 511a, Rio de Janeiro, 20550-900 Brazil; 4 Department of Geosciences and Geography, University of Helsinki, PO, Box 64, FI-00014 Helsinki, Finland; 5 Soil Ecosystems, Natural Resources Institute Finland, Latokartanonkaari 9, 00790 Helsinki, Finland; 6 Department of Built Environment, Aalto University, PO Box 11000, 00076 AALTO Espoo, Finland; 7 Centre for Biodiversity Genomics, University of Guelph, Guelph, ON Canada; 8 Department of Integrative Biology, University of Guelph, Guelph, ON Canada; 9 Department of Biological and Environmental Science, University of Jyväskylä, Jyväskylä, Finland; 10 Centre for Biodiversity Dynamics, Department of Biology, Norwegian University of Science and Technology, N-7491 Trondheim, Norway

**Keywords:** Community ecology, Microbial ecology

## Abstract

With anticipated expansion of agricultural areas for food production and increasing intensity of pressures stemming from land-use, it is critical to better understand how species respond to land-use change. This is particularly true for microbial communities which provide key ecosystem functions and display fastest responses to environmental change. However, regional land-use effects on local environmental conditions are often neglected, and, hence, underestimated when investigating community responses. Here we show that the effects stemming from agricultural and forested land use are strongest reflected in water conductivity, pH and phosphorus concentration, shaping microbial communities and their assembly processes. Using a joint species distribution modelling framework with community data based on metabarcoding, we quantify the contribution of land-use types in determining local environmental variables and uncover the impact of both, land-use, and local environment, on microbial stream communities. We found that community assembly is closely linked to land-use type but that the local environment strongly mediates the effects of land-use, resulting in systematic variation of taxon responses to environmental conditions, depending on their domain (bacteria vs. eukaryote) and trophic mode (autotrophy vs. heterotrophy). Given that regional land-use type strongly shapes local environments, it is paramount to consider its key role in shaping local stream communities.

## Introduction

Changes in land use are among the strongest drivers affecting species communities, diversity, and the ecosystem services they provide [1, 2]. Land-use changes do not only alter terrestrial ecosystems but also influence adjacent freshwater biodiversity and ecosystem functions in lakes and streams within the drainage systems [3, 4]. Freshwater ecosystems are thus considered sentinels to the impact of altered landscape processes since they are governed by the resulting changes in nutrient loads and exports of organic matter of vicinal areas [5]. Freshwater environments are particularly affected by the conversion from forested areas to agricultural lands through higher deposition of nutrients, dissolved organic matter, suspended solids and pollutants that alter water quality [6, 7]. At today’s numbers, about 38% of the ice-free land surface is dedicated to agricultural activities [8], and we have—by the year of 2022—crossed the milestone of 8 billion living humans on Earth. With the projected population growth to 10 billion by 2050 [9] we should expect human pressure for food production to expand agricultural areas and increase land use intensity [10] further affecting freshwater community structure and ecosystem functioning.

Community composition and biodiversity are widely used as surrogates to assess aquatic ecosystem status and functioning [11–13]. This is because water chemistry typically varies greatly even at short timeframes especially in smaller streams due to hydrological changes. Among several taxa, microbial communities emerge as a good bioindicator of fast environmental changes, due to their short generation times, large population densities and wide regional occurrence. They play an important role for ecosystem functioning by decomposing organic matter, transforming nutrients, and providing the basal food resource for higher trophic levels such as macroinvertebrates [14–16]. Accounting for the community structure of microbes has proven beneficial for explaining specific ecosystem functions [17]. Thus, taking a community perspective when investigating the impacts of land-use change on aquatic ecosystems is paramount. Most studies that investigate microbial community responses to environmental pressures commonly rely on ordination or cluster analyses that fail to account for potential species interactions and evolutionary constraints of environmental responses. During the last decade, species distribution modelling has become a common and fundamental tool to better understand and predict environmental filtering of communities [18]. Joint species distribution models (JSDM) in particular provide novel insight on community wide, yet taxa-specific responses with the advantages of taxa not being modelled independently but with an underlying joint structure related to abiotic (i.e., environment) and biotic filtering (i.e., species co-occurrences) simultaneously. Previous research on microbial stream communities has addressed the impacts of local environmental conditions [19, 20] and land-use types [21–23], including influences on biodiversity and function [24, 25]. However, the dependencies between regional land use type as a proxy variable and small-scale environmental conditions shaping communities are often neglected, leading to underestimated impacts of land use change. Using novel community level statistical advances provided by JSDM frameworks offers better understanding on the combined effects stemming from local environmental and regional land use pressures, and their respective contribution to microbial community composition and assembly processes. This is especially true as land-use impacts and changes in local environmental conditions in streams vary at different temporal scales, with land-use changes occurring at much longer time scales than any change in local stream conditions [3]. Even past land use is shown to influence water chemistry [26, 27]. Therefore, quantifying the extent of land-use effects on local environmental conditions will improve the understanding of microbial community patterns emerging from the interplay between regional land-use and local environmental conditions.

The goal of this study is to quantify the contribution of land-use types (forested vs. agricultural) in determining local environmental variables (i.e., water chemistry variables) and to uncover the impact of both, land-use, and environment, on microbial stream community assembly. With metabarcoding, we leverage bacteria and eukaryote community data from 120 stream sites in Finland to understand the resulting microbial community composition stemming from these drivers. Here we take advantage of a hierarchical JSDM to disentangle and quantify the species- and domain-specific (bacteria and eukaryote) drivers shaping community composition, while simultaneously accounting for phylogenetic constraints in species responses to the environment, as well as their trophic mode, i.e., being autotrophic or heterotrophic. This enables us to shed light on potential domain and/or trophy-specific responses to environmental conditions and susceptibility of different energy pathways. We further investigate the resulting impact of environmental filtering on microbial communities, using a region of common community profile approach.

## Material and methods

### Field sampling

**Fig. 1:**
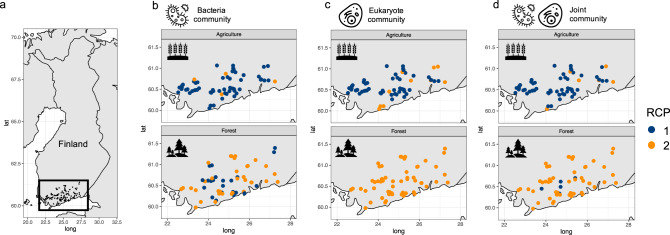
Study area and community profiles associated with land-use type. Map of (**a**) study area including sample region and sites, and (**b**–**d**) detailed sample sites with upper and lower panel including agricultural and forested sites, respectively, where colours represent the Region of Common Profile (RCP) based on community composition of (**b**) bacteria, (**c**) eukaryotes and (**d**) bacteria and eukaryotes.

Samples were collected in June–August 2020. In total, 120 stream sites were sampled from independent catchments totalling 60 agriculture and 60 forested sites (Fig. 1). The agriculture sites had >30% agricultural areas in the catchment area and the forested sites had <10% anthropogenic land use (i.e., agricultural areas + artificial surfaces) in the catchment area. The land cover and land use information were derived from the European land cover and land use classification CORINE (Coordination of information on the environment) dataset [28, 29] In the field, ten cobble-sized stones were randomly selected from different parts of each stream site for biofilm sampling. Biofilms were collected by scraping the surfaces of these stones (25 cm^2^ per stone) using a sponge (ca. 2 cm × 2 cm × 2 cm) and the resulting suspension was combined into a composite sample. At a few sites, less than ten stones were available. In these cases, multiple samples were collected from some larger stones so that the sampled stone surface area was the same for all sites. At each site, new sampling equipment were used, and samples were collected into sterilized sample containers. The samples were then stored frozen (−20 °C) and freeze-dried prior to subsequent laboratory analyses.

Simultaneously with biofilm sampling, water temperature, pH, and conductivity were measured using a Hach HQ40d multimeter (Hach, Loveland, CO, USA) and water samples were collected. The water samples were analyzed in the laboratory for total phosphorus according to SFS-EN ISO 6878 and for total nitrogen according to standard SFS-EN ISO 11905–1 with a Hach Lange DR 5000 spectrometer (Hach Lange GmbH, Düsseldorf, Germany).

### Environmental DNA and metabarcoding

DNA extraction, PCR and sequencing were performed at the Centre for Biodiversity Genomics, University of Guelph, Guelph, Canada. The lyophilized samples were sorted by weight into three categories (small, medium, and large). The volume of 1–6 mL of the Insect Lysis Buffer [30] with 10% Proteinase K was applied to the samples in proportion to their weight. The mixtures were vigorously vortexed, and 1 mL from each sample was subsampled into the Lysing matrix A, 2 mL tubes (MP Biomedicals) immediately. The lysates were ground in the TissueLyser at 30 Hz for 5 min, then incubated for 1 h at 56 °C, then frozen at −20° overnight, then incubated at 65 °C for 1 h 30 min. This procedure helped to break cellulous walls of the microscopic algae. DNA was extracted from the lysates with the modified automated DNA extraction protocol [31]. An additional precipitation on the Glass Fibre membrane with the consequent 5x dilution of DNA was used to obtain the best PCR result. Each sample was amplified in three replicas. The first round of PCR was performed with the following target-specific primers: Reuk454FWD1 (5'-CCAGCASCYGCGGTAATTCC-3') and V4r (5'-ACTTTCGTTCTTGAT-3') for eukaryotic 18 S rRNA region V4, ~380 base pairs; V8F (5'-ATAACAGGTCTGTGATGCCCT-3') and 1510 R (CCTTCYGCAGGTTCACCTAC) for eukaryotic 18 S rRNA regions V8-V9, ~330 base pairs; 341 F (5'-CCTACGGGNGGCWGCAG-3') and 805 R (5'-GACTACHVGGGTATCTAATCC-3') for bacterial 16 S rRNA regions V3-V4, ~460 base pairs. All primers were tailed with forward and reverse universal PacBio adaptors: PB1-forward (5'-GCAGTCGAACATGTAGCTGACTCAGGTCAC-3') and PB1-reverse (5'-TGGATCACTTGTGCAAGCATCACATCGTAG-3'). The following PCR1 conditions were used for 18 S V4 region: 94 °C for four minutes, followed by 35 cycles of 94 °C for 30 s, 46 °C for 30 s, 72 °C for 45 s, followed by 72 °C for ten minutes. The following PCR1 conditions were used for 18 S V8-V9 regions: 94 °C for four minutes, followed by 35 cycles of 94 °C for 30 s, 52 °C for 30 s, 72 °C for 45 s, followed by 72 °C for ten minutes. The following PCR1 conditions were used for 16 S V3-V4 regions: 94 °C for four minutes, followed by 35 cycles of 94 °C for 30 s, 50 °C for 30 s, 72 °C for 45 s, followed by 72 °C for ten minutes. PCR1 products were diluted with molecular grade ddH_2_0 in 1:1 ratio. The 1 µL of diluted PCR1 product was used as a template in the second PCR (PCR2). The PCR2 was performed in 384-well microplate with the total volume of the PCR reaction 6 µL, using forward and reverse barcoded universal PacBio primers Plate-384v2 kit, and following PCR conditions: 94 °C for two minutes, followed by 20 cycles of 94 °C for 40 s, 64 °C for one minute, 72 °C for one minute, followed by 72 °C for ten minutes. The resulting asymmetrically labelled amplicon products were pooled for DNA sequencing and quantified with Qubit 2.0 Fluorometer (ThermoFisher). SMRTbell libraries for each fragment were prepared using Express Template Prep Kit 2.0 with barcoded adapters bc1015_BAK8B_OA, bc1016_BAK8B_OA, bc1017_BAK8B_OA. Library QC was performed using Agilent 100 Bioanalyzer. Sequencing was performed using Sequel platform (Pacific Biosciences) on a single 1 M SMRTcell v3 with eight-hour movie time and 20pM on-plate loading concentration. The resulting polymerase reads were demultiplexed in SMRTLink v.8 to generate datasets for each amplicon fragment. By-strand circular consensus reads (CCS) were generated for each dataset with minimum predicted accuracy 99%. Final CCS reads were further demultiplexed for individual samples based on exact scoring of forward and reverse barcoded primers. Subsequent bioinformatic analysis involved removal of loci-specific primer sequences with *cutadapt* [32] and assigned the sequences into operational taxonomic groups using *vsearch* [33] with 97% similarity for both bacteria and eukaryotes. Taxonomic assignment of representative sequences for each OTU was done with the *classify*.*seqs* command at Mothur [34] against Silva 138 [35] for bacteria and pr2 version 4.14.0 [36].

With high number of singleton OTUs we used species level annotation for bacteria and order level for the analysis of eukaryotes. The bacterial taxa with domain unknown or the ones classified as Chloroplast were removed from the further analysis as were the eukaryal taxa with domain unknown or phyla/supergroup level annotated as *Eukaryota_unclassified*. We further joint taxa with same unknown classification level for either genus or order. Taxa that were present in more than 10 of the analyzed 120 samples were kept for the statistical analysis which were conducted with the presence-absence data. For phylogenomic analysis we constructed Newick-formatted phylogenetic trees with MEGAX [37] using MUSCLE nt alignment [38] and UPGMA clustering.

### Region of common community profile

We evaluated differences in community compositions and linked these to land use types by using a region of common profile (RCP) approach [39]. This was done by grouping sites based on their biological content, i.e., species occurrence data. Hence, communities within the same RCP are more similar in species composition than communities belonging to a different RCP. We used the *dist.binary* function from the *ade4* package *v*. *1.7-16* [40] in the R environment [41] to compute the Jaccard dissimilarity matrix for binary data using the raw presence-absence data including bacteria and eukaryote OTUs. Subsequently we used the *cascadeKM* function, a K-means partitioning using a range of values of K, here between 1 and 10, from the package *vegan v. 2.5-6* [42] to find the optimal number of clusters emerging from the community data using 1000 iterations. For optimal cluster evaluation, we used the well-established “calinski” criterion [43], included as output in the *cascadeKM* function. We used the resulting optimal data partition as RCPs, each reflecting sites of similar community composition.

### Joint species distribution modelling

Emerging advances in statistical community modelling frameworks now allow us to specifically quantify how species’ responses to environmental variation depend on traits and their phylogenetic relationships [44]. We used a hierarchical joint species distribution modelling (JSDM) approach, the Hierarchical Modelling of Species Communities (HMSC; [44–46]). HMSC can incorporate trait and phylogenetic data of communities to improve estimations of species’ responses to environmental covariates while also quantifying their contribution to the explained variation. First, to understand how land-use types and the spatial location of site within land use type influenced the local environmental variables, we build an environmental model *m*_*env*_, where we included water conductivity, pH, temperature [°C], total phosphorus concentration [ug/l], and the total nitrogen to phosphorus ratio as response variables with the explanatory variables being land-use type (agricultural vs. forested) as a fixed effect and the spatial location of sites, given as latitudinal and longitudinal coordinates, as the random effect. This enabled us to construct a causal flow diagram quantifying the explained variation of environmental covariates related to the spatial setting and land-use type. Prior to the analysis we applied a log-transformation to the response variables water conductivity; phosphorus concentration and the N:P ratio; to better approximate normal distributions.

Subsequently, we constructed three different community models based on, (1) the bacterial community *m*_*bac*_, (2) the eukaryotic community *m*_*euk*_, and (3) the joint community *m*_*joint*_ including both domains, bacteria, and eukaryotes. We modelled spatially explicit eukaryotic and bacterial occurrences probabilities, based on OTUs, and their inference with environmental parameters. Our response variables were 217 OTUs, including 58 bacteria and 159 eukaryote taxa. The first two models, *m*_*bac*_ *and m*_*euk*_, allowed us to quantify domain specific environmental drivers and the degree to which occurrence probabilities of included taxa depend on evolutionary constraints based on their phylogenetic relationship. With *m*_*joint*_ we modelled cross-domain species occurrences, specifically accounting for trophic modes within the community and investigated common community profiles in relation to land-use types. Each model was fitted with the same environmental variables, and the same spatially explicit structure. We included land-use, water conductivity, pH, temperature, total phosphorus concentration, and the total nitrogen to phosphorus ratio (N:P) as fixed effects. To account for the spatial structure of the study design, we included a spatially explicit random effect at the level of site, represented by latitudinal and longitudinal coordinates. Prior to the community model analyses, phosphorus concentrations as well as the N:P ratio were log-transformed.

HMSC incorporates a hierarchical layer accounting for phylogenetic constraints and traits in species responses to environmental variables [47]. In *m*_*bac*_ *and m*_*euk*_ we included the phylogenetic structure of the community as well as the categorical trait *trophy* accounting for autotrophic and heterotrophic species. We evaluated the strength of a potential phylogenetic signal to environmental variables within communities, i.e., if closely related species tend to have similar directional responses to environmental variables. The phylogenetic correlation parameter is denoted as *rho*, with possible values between 0 and 1 indicating whether the residual variance among the species is independent (0) or if their environmental responses are fully structured by their phenology (1). For *m*_*joint*_ we did not include phylogenetic information due to the cross-domain analysis but instead, in addition to the trait *trophy*, we also included the trait *domain*, enabling us to account for domain-specific trophic modes. We then use *m*_*joint*_-specific parameters to quantify the domain- and trophy-specific environmental responses of taxa within the joint community.

We fitted all four above described models (*m*_*env*_*, m*_*bac*_*, m*_*euk*_ and *m*_*joint*_) with the Hierarchical Modelling of Species Communities (HMSC) R package *Hmsc* [46]. Since *m*_*bac*_, *m*_*euk*_ and *m*_*joint*_ are based on binary presence absence data, the models followed a Bernoulli distribution with a *probit* link function, while *m*_*env*_ followed a Gaussian distribution with an *identity* link function. We sampled the posterior distribution of each model with four Markov Chain Monte Carlo (MCMC) chains, each of which was run for 37,500 iterations, of which the first 12,500 were discarded as burn in. The chains had a thinning of 100 to yield 250 posterior samples per chain, resulting in 1000 posterior samples per model in total. We subsequently assessed MCMC convergence by examining the potential scale reduction factors [48] of the model parameters.

To examine the explanatory power of the models, we evaluated OTU-specific Tjur R^2^ [49] and AUC [50] values for the binary presence-absence models *m*_*bac*_, *m*_*euk*_ and *m*_*joint*_, and R^2^ values for *m*_*env*_. We quantified the drivers of community structure following Ovaskainen et al. [45] to partition explained variation among the fixed and random effects.

## Results

### Sequence data obtained

For bacteria a total of 378 (from 295–523) and for eukaryotes a total of 363 (from 85–865) PacBio Sequel sequence reads per sample were used in the analysis. The by-strand circular consensus reads (CCS) were generated for each dataset with a minimum predicted accuracy of 99%. With high quality due to the short read length and several cycles of sequencing, we used all reads in the analysis.

### Land-use type structures community profiles at local environment

The data partitioning to highlight sites with common community profiles resulted in two optimal clusters for each domain, bacteria, and eukaryotes, as well as their joint composition (Fig. S1), namely RCP 1 and RCP 2 (Fig. 1b–d). We found strong separations between common community profiles depending on land-use type, where RCP 1 was strongly associated with agricultural, and RCP 2 with forested areas.

**Fig. 2:**
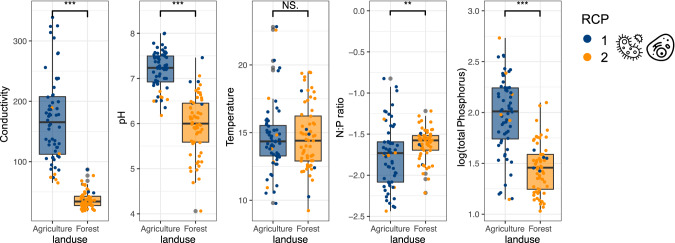
Comparison of environmental conditions at land-use types. Presence of joint bacteria and eukaryote community RCPs in relation to realized environmental conditions at land-use types.

Local environmental conditions showed statistically significant differences between land-use types for all included environmental variables except temperature (Fig. 2). Linking the realized RCPs to the local environmental conditions at land-use types, we found that RCP 1 with high prevalence in agricultural areas was associated with higher values in water conductivity, pH, and phosphorus concertation compared to RCP 2 with high prevalence at forested sites (Fig. 2). The nitrogen to phosphorus ratio was on average higher in forested areas. Water temperature did not differ between land-use types.

### Model convergence and fit

The MCMC convergence of all four models (*m*_*env*_*, m*_*bac*_*, m*_*euk*_ and *m*_*joint*_) was good, indicated by the potential scale reduction factor being ≪ 1.1 (Table 1). The models showed a satisfactory fit indicated by discriminatory and explanatory powers (Table 1).

**Table 1:** Model diagnostics for the environmental model *m*_*env*_ as well as the three community models *m*_*bac*_ (bacteria), *m*_*euk*_ (eukaryotes) and *m*_*joint*_ (bacteria and eukaryotes).

	psrf		mean discriminatory/explanatory power		
	mean point est.	upper C.I.	Tjur R^2^	AUC	R^2^
*m*_*env*_	1.004	1.015			0.690
*m*_*bac*_	1.002	1.008	0.111	0.785	
*m*_*euk*_	1.002	1.009	0.095	0.760	
*m*_*joint*_	1.003	1.012	0.096	0.772	

The Gelman-Rubin potential scale reduction factor (psrf) as measure for MCMC convergence being ≪ 1.1 indicates good conversion for all models. Explanatory power of *m*_*env*_ is measured via R^2^, while the community models, based on presence absence data, are evaluated by the coefficient of discrimination Tjur R^2^ and area under the curve AUC for their discriminatory power.

### Variance partitioning of land-use, local environment, and community relationships

**Fig. 3:**
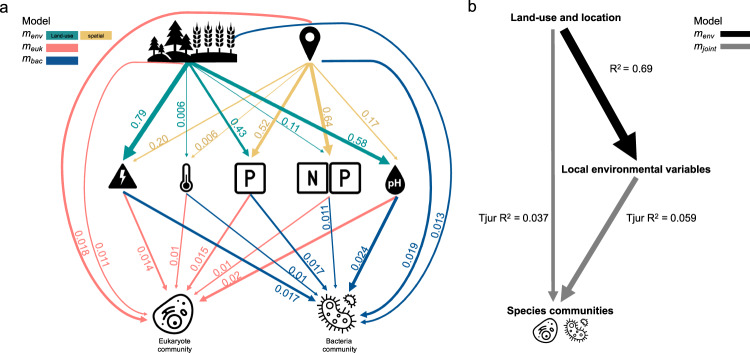
Causal flow diagrams summarizing the relationships between land-use type, environmental variables, and species communities in terms of explained variation. **a** Detailed causal flow diagram indicating explained variation (R^2^) of environmental variables by land-use type (top row left, forest vs. agriculture) and explicit spatial location (top row right). Thickness of arrows correspond to indicated R^2^ values. Middle row: symbols from left to right represent conductivity, water temperature, total phosphorus concentration, total nitrogen to phosphorus ratio, and pH with respective Tjur R^2^ values in explaining eukaryotic (*m*_*euk*_ bottom row left) and bacterial (*m*_*bac*_ bottom row right) species occurrences at the community level. Note that arrow thickness is proportional among Tjur R^2^ values (red and blue, *m*_*euk*_ *and m*_*bac*_ respectively) and among R^2^ values (green and yellow, *m*_*env*_). **b** Summarized causal flow diagram indicating mean explained variation of environmental variables by land-use type and location (R^2^ from *m*_*env*_, black), and fractions of explained variation of land-use and location as well as environmental variables on the joint community (Tjur R^2^ from *m*_*joint*_, grey).

We found that land-use type and sample location are strong predictors for local environmental conditions, as indicated by the respective R^2^ values of *m*_*env*_ (Fig. 3). This is especially true for water conductivity and total phosphorus concentrations, where land-use and location together explained 98.5% and 94.7% of their total variation, respectively. The explanatory power of land-use type was particularly strong for conductivity (79%), pH (58%), and total phosphorus concentration (43%), while the explicit spatial effect contributed most to explained variation in nutrient concentrations, i.e., the nitrogen to phosphorus ratio (64%) as well as total phosphorus concentrations (52%) in streams. Only the variation of water temperature was independent of land-use type and location with <1% explained variation, respectively.

For both, eukaryote (*m*_*euk*_) and bacteria (*m*_*bac*_) communities, taxa occurrences were influenced similarly, in terms of proportions of the explained variation by the fixed and random effects (Fig. 3a). Water conductivity, phosphorus concentration, and pH were the strongest predictors, each alone exceeding the effect of land-use type as proxy variable as fixed effect. However, the spatial random effect had the second strongest explanatory power for species occurrences after pH, in both models, *m*_*euk*_ and *m*_*bac*_.

When considering all eukaryotic and bacterial taxa together as joint community (*m*_*joint*_), we found that of the total explained variation in species occurrences, about two thirds (61.9%) can be attributed to the local environmental conditions, while effects of the proxy variable land-use type (14.1%) and the explicit spatial location (24%) only accounted for one third of variation combined (Figs. 3b, S2). However, roughly 70% of the variation in local environmental variables can be attributed to land-use and location (Fig. 3b).

### Species-environment responses depend on domain-specific trophic modes

**Fig. 4:**
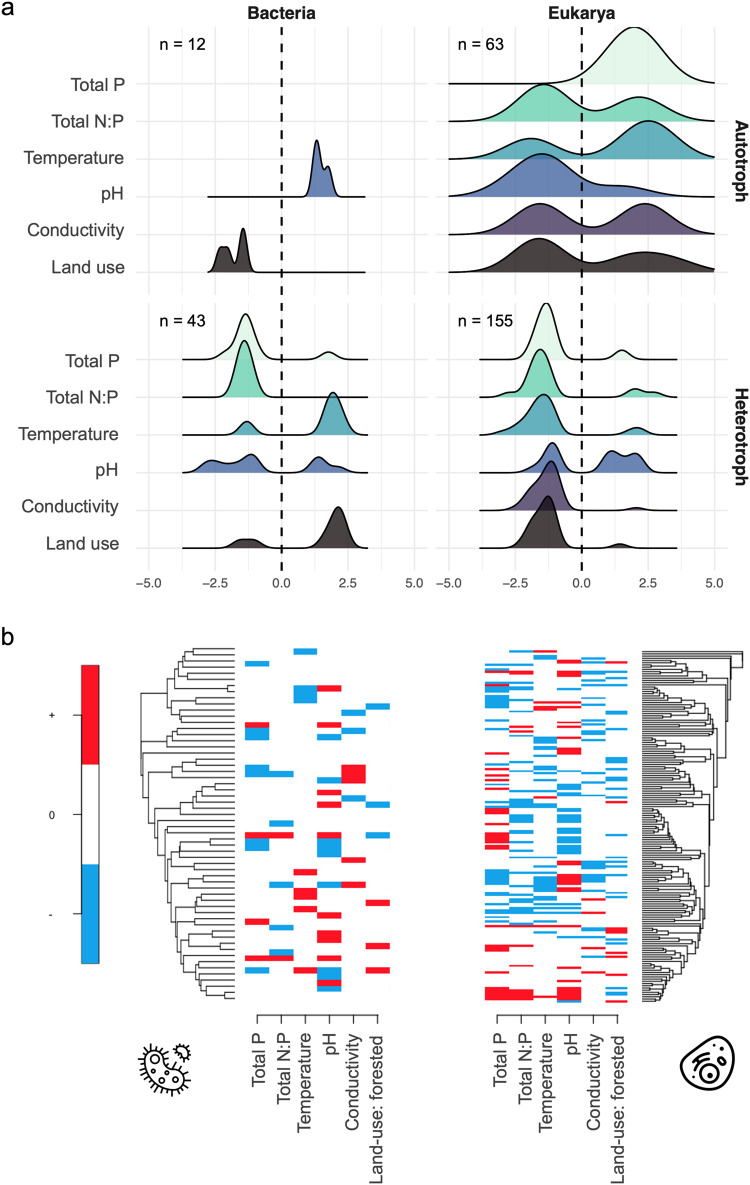
Responses of species to environmental variables and land use. **a** Density distribution of domain- and trophy- specific environment relationships with statistical support of 90% posterior probability, illustrated by standardized variable effects from *m*_*join*t_. The number of statistically supported responses of taxa per panel group is indicated by *n*. **b** Phylogenetically structured directional responses of taxa occurrences to variables (left *m*_*bac*_, right *m*_*euk*_). Responses that are positive with at least 90% posterior probability are shown by red, responses that are negative with at least 90% posterior probability are shown in blue. Responses that did not gain strong statistical support are shown in white. Tree tip labels and taxonomic classifications are given in Supplementary Tables S1, S2 and Figs. S2, S3.

Using the joint community model (*m*_*joint*_) to account for domain-specific response types to environmental variables, we found that the effects of environmental conditions differed across bacteria and eukaryotes as well as across autotrophs and heterotrophs (Fig. 4). Among all autotrophic eukaryotes, individual taxa showed both positive and negative responses to the local and regional variables, except for total phosphorus concentrations, which generally increased the probability of taxon occurrence. Conversely, heterotrophic eukaryotes that showed statistically supported responses were mainly negatively associated with the included variables (Fig. 4a). While autotrophic bacteria were more likely to occur at higher pH values and in agricultural than in forested areas, the heterotrophic bacteria were less likely to occur in nutrient rich waters, being more favoured by warmer waters in forested areas (Fig. 4a). Noteworthy, there was a moderately strong phylogenetic signal in both bacteria (*rho* = 0.71, SD = 0.07) and eukaryote communities (*rho* = 0.75, SD = 0.08), suggesting that closely related species in each community tended to respond similarly to environmental variables (Fig. 4b).

## Discussion

Land-use types have a profound effect on freshwater communities, their biodiversity and on the ecosystem functions they provide [1, 51]. However, our results underline that regional land-use effects determine microbial community assembly by primarily shaping local environmental conditions. Here we demonstrated that (i) microbial stream community composition is closely linked to land-use type, (ii) local environmental conditions, driving microbial species responses, mediate the effects of land-use on microbial communities, and (iii) taxon responses show systematic variation to environmental variables, depending on their domain (bacteria vs. eukaryote) and trophic mode (autotrophy vs. heterotrophy).

We found a clear differentiation between the community compositions depending on land use type. This was true not only for the bacterial community but also for the eukaryotic community as well as for their joint composition. For each of the three community types two regions of common community profile (RCP) emerged, each reflecting similarity in community composition with affinities to either agricultural or forested areas, without any *a priori* assumptions regarding environmental conditions at the respective sites. This clear separation beyond taxonomic domains, suggests that environmental filtering and species sorting are the main processes underpinning the microbial community structure [52–54]. Different land use type-associated community composition also point to differences in water quality and microbial activity with altered stream metabolism possibly affecting higher trophic levels through altered food web structure [14, 51]. The strong observed impact of environmental filtering on the microbial meta-community supports their potential as bioindicators for the assessment of environmental conditions [55].

The effects of regional land-use on the local environmental conditions (e.g., water physico-chemistry) are often overlooked when investigating land use effects on communities [21, 56]. This may owe to the common aim to disentangle the respective contribution of land use, such as urbanized, agricultural, or forested areas, on species or communities in a certain environmental setting. However, here we show that regional land-use types overwhelmingly determine local key environmental drivers of microbial stream communities. While our results demonstrate that land use, when considered as large-scale proxy variable, explained less variation of species occurrences than local environmental conditions, the effects of land use on the variation of local stream conditions was substantial. This suggests that regional land use effects determine local stream conditions that in turn are the main driver of microbial stream community assembly. We argue that the full impact of land use may be underestimated when failing to consider that local environmental conditions mediate regional land use effects. The strongest environmental drivers explaining community composition, were also those which were best explained by land-use types, namely, water conductivity, water pH and phosphorus concertation, with statistically significant differences between agricultural and forested areas. However, we acknowledge that while our included explanatory covariates do reflect general parameters of local water quality, we did not consider any organic matter compositions here, that may have added a more detailed understanding in the mechanisms of community structures between land use types. Water pH-levels have been previously demonstrated to be a major driver of microbial stream communities [57, 58]. Our results support this finding with pH having the highest explanatory power of the fixed effects in all three community models, *m*_*euk*_*, m*_*bac*_ *and m*_*joint*_. We found high levels of pH values and water conductivity to be associated with agricultural land use. Microbial community responses to elevated values in both, pH and conductivity, have been linked to increased respiration rates, indicating stress responses, with the potential to destabilize microbial ecosystem function [51].

Although we found consistent responses of communities belonging to different domains, i.e., eukaryote vs. bacterial community, as well as the joint cross domain community, we found systematic variation of species responses to environmental variables and land-use type, depending on domain-specific trophic modes. Autotrophic eukaryotes show mainly positive associations to phosphorus concentration, while displaying a bimodal distribution of both negative and positive responses of included taxa to the remaining environmental variables, including land use type, suggesting that this group of species comprises members with a heterogeneous range of environmental niches. Heterotrophic eukaryotes on the other hand seemed more constraint in their niche space, showing mainly negative responses to realized environmental variables. While heterotrophic bacteria exhibited similar negative responses to nutrient concentrations as their eukaryotic counterparts, they had opposite responses to water temperature and land-use type, and no detected association with water conductivity. This indicates that heterotrophic bacteria and eukaryotes are associated with different environmental conditions, likely excluding competitive interactions as well as environment-specific energy flows. The fact that autotrophic bacteria only showed statistically supported responses to pH and land use points towards their robustness to environmental drivers and wide-ranging niche space. These trophy-specific responses are supported by the detected signal of phylogenetically structured environmental responses in both communities, signifying phylogenetic niche conservatism. When dividing responses of energy flow pathways into ‘green’, autotroph-based, and ‘brown’, heterotroph-based [59, 60], our results suggest that the brown pathways are more susceptible to environmental change and land use types than the green. Depending on the environmental setting as well as on primary consumer preference, shifts in such energy flow pathways may have ecosystem wide implications. Highlighting these different domain and trophy specific response types at the community level, and revealing their potential influence on ecosystem function, is not possible by applying the commonly used statistical tools such as ordination analyses, but requires the use of more timely advances in community ecology such as JSDM. Considering that land-use type is a strong determinant of the local environment, it will be important to not only consider its impact on species communities as a large-scale proxy variable, but also the even stronger local impact through mediated environmental conditions.

Our findings challenge the common approach to consider impacts of land-use change and local environmental variables on microbial communities independently from each other. We show that most of the explained variation in species responses is attributed to the local conditions, which are in fact to the largest part determined by regional land-use types. This strong impact of land-use on community assembly across domains redefines our current understanding towards the expanding pressures stemming from land-use change, provides better understanding to community wide responses, and may ultimately support stream ecosystem conservation efforts.

## Supplementary Information

Supplementary information

## Data Availability

Raw sequence and metadata can be found in the European Nucleotide Archive (ENA) under the project PRJEB62688.
