# Supplementary material for: Local eukaryotic and bacterial stream community assembly is shaped by regional land use effects
Source: ISME Commun. 2023 Jun 26;3:65. doi: 10.1038/s43705-023-00272-2 (PMC10293236; doi:10.1038/s43705-023-00272-2)
Supplement: Supplementary file 1 — Supplementary information [file 43705_2023_272_MOESM1_ESM.pdf]

Supplementary information Tables and Figures to

Local eukaryotic and bacterial stream community assembly is shaped by regional land use effects

Benjamin Weigel[1,2*], Caio Graco-Roza[3,4], Jenni Hultman[5], Virpi Pajunen[4,6], Anette Teittinen[4], Maria Kuzmina[7], Evgeny V. Zakharov[7,8], Janne Soininen[4, ‡], Otso Ovaskainen[1, 9, 10, ‡]

[1] Research Centre for Ecological Change, Organismal and Evolutionary Biology Research Programme, Faculty of Biological and Environmental Sciences, University of Helsinki, P.O. 65, Helsinki FI-00014, Finland

[2] INRAE, EABX, 50 avenue de Verdun, 33612 Cestas, France

[3] Laboratory of Ecology and Physiology of Phytoplankton, Department of Plant Biology, State University of Rio de Janeiro, Rua São Francisco Xavier 524, PHLC, Sala 511a, 20550-900 Rio de Janeiro, Brazil

[4] Department of Geosciences and Geography, University of Helsinki, PO, Box 64, FI-00014 Helsinki, Finland

[5] Natural Resources Institute Finland, Latokartanonkaari 9, 00790 Helsinki, Finland

[6] Department of Built Environment, Aalto University, PO Box 11000, 00076 AALTO, Espoo, Finland

[7] Centre for Biodiversity Genomics, University of Guelph, Guelph, Canada

[8] Department of Integrative Biology, University of Guelph, Guelph, Canada

[9] Department of Biological and Environmental Science, University of Jyväskylä, Jyväskylä, Finland

[10] Centre for Biodiversity Dynamics, Department of Biology, Norwegian University of Science and Technology, N-7491 Trondheim, Norway

** Corresponding author: benjamin.weigel@helsinki.fi, benjamin.weigel@inrae.fr*

‡ These authors jointly supervised this work.

Table S1: Bacteria taxonomic structuring following the phylogenetic tree in Fig. S3 and S4b

Domain	Phylum	Class	Order	Family	Genus
Bacteria	Proteobacteria	Alphaproteobacteria	Alphaproteobacteria_unclassified	Alphaproteobacteria_unclassified	Alphaproteobacteria_unclassified
Bacteria	Proteobacteria	Alphaproteobacteria	Rhizobiales	Rhizobiales_unclassified	Rhizobiales_unclassified
Bacteria	Proteobacteria	Alphaproteobacteria	Rhizobiales	Xanthobacteraceae	Xanthobacteraceae_unclassified
Bacteria	Proteobacteria	Alphaproteobacteria	Sphingomonadales	Sphingomonadaceae	Sphingomonadaceae_unclassified
Bacteria	Proteobacteria	Alphaproteobacteria	Caulobacterales	Caulobacteraceae	Caulobacteraceae_unclassified
Bacteria	Proteobacteria	Alphaproteobacteria	Acetobacterales	Acetobacteraceae	Acetobacteraceae_unclassified
Bacteria	Proteobacteria	Proteobacteria_unclassified	Proteobacteria_unclassified	Proteobacteria_unclassified	Proteobacteria_unclassified
Bacteria	Firmicutes	Firmicutes_unclassified	Firmicutes_unclassified	Firmicutes_unclassified	Firmicutes_unclassified
Bacteria	Proteobacteria	Gammaproteobacteria	Methylococcales	Methylomonadaceae	Methylomonadaceae_unclassified
Bacteria	Proteobacteria	Gammaproteobacteria	Gammaproteobacteria_unclassified	Gammaproteobacteria_unclassified	Gammaproteobacteria_unclassified
Bacteria	Proteobacteria	Gammaproteobacteria	Aeromonadales	Aeromonadaceae	Aeromonas
Bacteria	Proteobacteria	Gammaproteobacteria	Enterobacterales	Enterobacterales_unclassified	Enterobacterales_unclassified
Bacteria	Proteobacteria	Gammaproteobacteria	Diplorickettsiales	Diplorickettsiaceae	Rickettsiella
Bacteria	Proteobacteria	Gammaproteobacteria	Pseudomonadales	Pseudomonadaceae	Pseudomonas
Bacteria	Bacteroidota	Bacteroidia	Chitinophagales	Chitinophagales_unclassified	Chitinophagales_unclassified
Bacteria	Proteobacteria	Gammaproteobacteria	Burkholderiales	Methylophilaceae	Methylophilaceae_unclassified
Bacteria	Proteobacteria	Gammaproteobacteria	Burkholderiales	Burkholderiales_unclassified	Burkholderiales_unclassified
Bacteria	Proteobacteria	Gammaproteobacteria	Burkholderiales	Burkholderiaceae	Polynucleobacter
Bacteria	Actinobacteriota	Actinobacteriota_unclassified	Actinobacteriota_unclassified	Actinobacteriota_unclassified	Actinobacteriota_unclassified
Bacteria	Actinobacteriota	Actinobacteria	Actinobacteria_unclassified	Actinobacteria_unclassified	Actinobacteria_unclassified
Bacteria	Actinobacteriota	Actinobacteria	Micrococcales	Micrococcales_unclassified	Micrococcales_unclassified
Bacteria	Myxococcota	Polyangia	Polyangiales	Polyangiaceae	Polyangiaceae_unclassified
Bacteria	Myxococcota	Myxococcia	Myxococcales	Anaeromyxobacteraceae	Anaeromyxobacter
Bacteria	Acidobacteriota	Acidobacteriae	Acidobacteriae_unclassified	Acidobacteriae_unclassified	Acidobacteriae_unclassified
Bacteria	Acidobacteriota	Acidobacteriota_unclassified	Acidobacteriota_unclassified	Acidobacteriota_unclassified	Acidobacteriota_unclassified
Bacteria	Acidobacteriota	Acidobacteriae	Acidobacteriales	Acidobacteriales_unclassified	Acidobacteriales_unclassified
Bacteria	Acidobacteriota	Acidobacteriae	Acidobacteriales	Acidobacteriaceae_	Acidobacteriaceae__unclassified
Bacteria	Firmicutes	Bacilli	Bacilli_unclassified	Bacilli_unclassified	Bacilli_unclassified
Bacteria	Armatimonadota	Fimbriimonadia	Fimbriimonadales	Fimbriimonadaceae	Fimbriimonadaceae_ge
Bacteria	Acidobacteriota	Blastocatellia	Blastocatellales	Blastocatellaceae	Blastocatellaceae_unclassified
Bacteria	Proteobacteria	Gammaproteobacteria	Burkholderiales	Comamonadaceae	Comamonadaceae_unclassified
Bacteria	Cyanobacteria	Cyanobacteriia	Leptolyngbyales	Leptolyngbyaceae	uncultured
Bacteria	Cyanobacteria	Cyanobacteriia	Cyanobacteriales	Xenococcaceae	Pleurocapsa_PCC-7319
Bacteria	Cyanobacteria	Cyanobacteriia	SepB-3	SepB-3_fa	SepB-3_ge
Bacteria	Cyanobacteria	Cyanobacteriia	Cyanobacteriia_unclassified	Cyanobacteriia_unclassified	Cyanobacteriia_unclassified
Bacteria	Cyanobacteria	Cyanobacteria_unclassified	Cyanobacteria_unclassified	Cyanobacteria_unclassified	Cyanobacteria_unclassified
Bacteria	Verrucomicrobiota	Verrucomicrobiae	Pedosphaerales	Pedosphaeraceae	Pedosphaeraceae_unclassified
Bacteria	Verrucomicrobiota	Verrucomicrobiae	Verrucomicrobiae_unclassified	Verrucomicrobiae_unclassified	Verrucomicrobiae_unclassified
Bacteria	Verrucomicrobiota	Verrucomicrobiae	Verrucomicrobiales	Rubritaleaceae	Luteolibacter
Bacteria	Verrucomicrobiota	Verrucomicrobiota_unclassified	Verrucomicrobiota_unclassified	Verrucomicrobiota_unclassified	Verrucomicrobiota_unclassified
Bacteria	Patescibacteria	Parcubacteria	Parcubacteria_unclassified	Parcubacteria_unclassified	Parcubacteria_unclassified
Bacteria	Planctomycetota	Planctomycetes	Pirellulales	Pirellulaceae	Pirellulaceae_unclassified
Bacteria	Planctomycetota	Planctomycetes	Planctomycetes_unclassified	Planctomycetes_unclassified	Planctomycetes_unclassified
Bacteria	Planctomycetota	Planctomycetes	Planctomycetales	Schlesneriaceae	Schlesneriaceae_unclassified
Bacteria	Planctomycetota	Planctomycetes	Planctomycetales	Planctomycetales_unclassified	Planctomycetales_unclassified
Bacteria	Bacteroidota	Bacteroidia	Cytophagales	Cytophagales_unclassified	Cytophagales_unclassified
Bacteria	Bacteroidota	Bacteroidota_unclassified	Bacteroidota_unclassified	Bacteroidota_unclassified	Bacteroidota_unclassified
Bacteria	Bacteroidota	Bacteroidia	Cytophagales	Hymenobacteraceae	Hymenobacter
Bacteria	Bacteroidota	Bacteroidia	Cytophagales	Spirosomaceae	Lacihabitans
Bacteria	Bacteroidota	Bacteroidia	Cytophagales	Spirosomaceae	Emticicia
Bacteria	Bacteroidota	Bacteroidia	Flavobacteriales	Flavobacteriaceae	Flavobacterium
Bacteria	Bacteroidota	Bacteroidia	Flavobacteriales	Flavobacteriaceae	Flavobacteriaceae_unclassified
Bacteria	Bacteroidota	Bacteroidia	Sphingobacteriales	env.OPS_17	env.OPS_17_ge
Bacteria	Bacteroidota	Bacteroidia	Sphingobacteriales	Sphingobacteriales_unclassified	Sphingobacteriales_unclassified
Bacteria	Bacteroidota	Bacteroidia	Bacteroidia_unclassified	Bacteroidia_unclassified	Bacteroidia_unclassified
Bacteria	Bacteroidota	Bacteroidia	Chitinophagales	Chitinophagaceae	Chitinophagaceae_unclassified
Bacteria	Bacteroidota	Bacteroidia	Sphingobacteriales	Sphingobacteriaceae	Sphingobacteriaceae_unclassified
Bacteria	Bacteroidota	Bacteroidia	Flavobacteriales	Flavobacteriales_unclassified	Flavobacteriales_unclassified

Table S2: Eukaryote taxonomic structuring following the phylogenetic tree in Fig. S4 and 4b

Domain	Supergroup	Division	Class	Order	Family	Genus	Species
Eukaryota	Stramenopiles	Ochrophyta	Bacillariophyta	Bacillariophyta_X	Raphid-pennate	Eunotia	Eunotia_pectinalis
Eukaryota	Stramenopiles	Ochrophyta	Bacillariophyta	Bacillariophyta_X	Raphid-pennate	Nitzschia	Nitzschia_linearis
Eukaryota	Stramenopiles	Ochrophyta	Bacillariophyta	Bacillariophyta_X	Raphid-pennate	Nitzschia	Nitzschia_unclassified
Eukaryota	Stramenopiles	Ochrophyta	Bacillariophyta	Bacillariophyta_X	Raphid-pennate	Surirella	Surirella_sp.
Eukaryota	Stramenopiles	Ochrophyta	Bacillariophyta	Bacillariophyta_X	Raphid-pennate	Surirella	Surirella_unclassified
Eukaryota	Stramenopiles	Ochrophyta	Bacillariophyta	Bacillariophyta_X	Raphid-pennate	Navicula	Navicula_unclassified
Eukaryota	Stramenopiles	Ochrophyta	Bacillariophyta	Bacillariophyta_X	Raphid-pennate	Navicula	Navicula_gregaria
Eukaryota	Stramenopiles	Ochrophyta	Bacillariophyta	Bacillariophyta_X	Araphid-pennate	Diatoma	Diatoma_tenue
Eukaryota	Stramenopiles	Ochrophyta	Bacillariophyta	Bacillariophyta_X	Araphid-pennate	Araphid-pennate_unclassified	Araphid-pennate_unclassified
Eukaryota	Stramenopiles	Ochrophyta	Bacillariophyta	Bacillariophyta_X	Raphid-pennate	Achnanthidium	Achnanthidium_minutissimum
Eukaryota	Stramenopiles	Ochrophyta	Bacillariophyta	Bacillariophyta_X	Raphid-pennate	Frustulia	Frustulia_gondwana
Eukaryota	Stramenopiles	Ochrophyta	Bacillariophyta	Bacillariophyta_X	Raphid-pennate	Eunotia	Eunotia_sp.
Eukaryota	Stramenopiles	Ochrophyta	Bacillariophyta	Bacillariophyta_X	Raphid-pennate	Pinnularia	Pinnularia_unclassified
Eukaryota	Stramenopiles	Ochrophyta	Bacillariophyta	Bacillariophyta_X	Raphid-pennate	Raphid-pennate_unclassified	Raphid-pennate_unclassified
Eukaryota	Stramenopiles	Ochrophyta	Bacillariophyta	Bacillariophyta_X	Raphid-pennate	Gomphonema	Gomphonema_unclassified
Eukaryota	Stramenopiles	Ochrophyta	Bacillariophyta	Bacillariophyta_X	Raphid-pennate	Gomphonema	Gomphonema_acuminatum
Eukaryota	Stramenopiles	Ochrophyta	Bacillariophyta	Bacillariophyta_X	Raphid-pennate	Gomphonema	Gomphonema_parvulum
Eukaryota	Stramenopiles	Ochrophyta	Bacillariophyta	Bacillariophyta_X	Raphid-pennate	Gomphonema	Gomphonema_angustatum
Eukaryota	Stramenopiles	Ochrophyta	Bacillariophyta	Bacillariophyta_X	Raphid-pennate	Eunotia	Eunotia_unclassified
Eukaryota	Stramenopiles	Ochrophyta	Chrysophyceae	Chrysophyceae_X	Chrysophyceae_Clade-B2	Naegeliella	Naegeliella_flagellifera
Eukaryota	Stramenopiles	Ochrophyta	Chrysophyceae	Chrysophyceae_X	Chrysophyceae_X_unclassified	Chrysophyceae_X_unclassified	Chrysophyceae_X_unclassified
Eukaryota	Stramenopiles	Ochrophyta	Ochrophyta_unclassified	Ochrophyta_unclassified	Ochrophyta_unclassified	Ochrophyta_unclassified	Ochrophyta_unclassified
Eukaryota	Stramenopiles	Ochrophyta	Chrysophyceae	Chrysophyceae_X	Chrysophyceae_Clade-D	Chrysophyceae_Clade-D_X	Chrysophyceae_Clade-D_X_sp.
Eukaryota	Stramenopiles	Ochrophyta	Chrysophyceae	Chrysophyceae_X	Chrysophyceae_Clade-C	Spumella	Spumella_unclassified
Eukaryota	Stramenopiles	Ochrophyta	Chrysophyceae	Chrysophyceae_X	Chrysophyceae_Clade-C	Chrysophyceae_Clade-C_unclassified	Chrysophyceae_Clade-C_unclassified
Eukaryota	Stramenopiles	Ochrophyta	Bacillariophyta	Bacillariophyta_X	Raphid-pennate	Surirella	Surirella_angusta
Eukaryota	Stramenopiles	Pseudofungi	Oomycota	Oomycota_X	Oomycota_X_unclassified	Oomycota_X_unclassified	Oomycota_X_unclassified
Eukaryota	Stramenopiles	Stramenopiles_unclassified	Stramenopiles_unclassified	Stramenopiles_unclassified	Stramenopiles_unclassified	Stramenopiles_unclassified	Stramenopiles_unclassified
Eukaryota	Stramenopiles	Pseudofungi	Oomycota	Oomycota_X	Peronosporales	Peronosporales_unclassified	Peronosporales_unclassified
Eukaryota	Stramenopiles	Pseudofungi	Oomycota	Oomycota_X	Saprolegniales	Saprolegniales_unclassified	Saprolegniales_unclassified
Eukaryota	Archaeplastida	Rhodophyta	Florideophyceae	Batrachospermales	Batrachospermales_X	Batrachospermales_X_unclassified	Batrachospermales_X_unclassified
Eukaryota	Archaeplastida	Rhodophyta	Florideophyceae	Batrachospermales	Batrachospermales_X	Batrachospermum	Batrachospermum_boryanum
Eukaryota	Archaeplastida	Rhodophyta	Florideophyceae	Batrachospermales	Batrachospermales_X	Sirodotia	Sirodotia_delicatula
Eukaryota	Archaeplastida	Rhodophyta	Florideophyceae	Batrachospermales	Batrachospermales_X	Batrachospermum	Batrachospermum_gelatinosum
Eukaryota	Stramenopiles	Ochrophyta	Bacillariophyta	Bacillariophyta_X	Radial-centric-basal-Coscinodiscophyceae	Melosira	Melosira_varians
Eukaryota	Stramenopiles	Ochrophyta	Bacillariophyta	Bacillariophyta_X	Bacillariophyta_X_unclassified	Bacillariophyta_X_unclassified	Bacillariophyta_X_unclassified
Eukaryota	Alveolata	Ciliophora	Spirotrichea	Euplotia	Aspidiscidae	Aspidisca	Aspidisca_sp.
Eukaryota	Alveolata	Ciliophora	Spirotrichea	Spirotrichea_unclassified	Spirotrichea_unclassified	Spirotrichea_unclassified	Spirotrichea_unclassified
Eukaryota	Opisthokonta	Fungi	Chytridiomycota	Chytridiomycotina	Chytridiomycetes	Chytridiomycetes_unclassified	Chytridiomycetes_unclassified
Eukaryota	Opisthokonta	Fungi	Chytridiomycota	Chytridiomycotina	Chytridiomycetes	Rhyzophidiales_X	Rhyzophidiales_X_sp.
Eukaryota	Opisthokonta	Fungi	Fungi_unclassified	Fungi_unclassified	Fungi_unclassified	Fungi_unclassified	Fungi_unclassified
Eukaryota	Alveolata	Alveolata_unclassified	Alveolata_unclassified	Alveolata_unclassified	Alveolata_unclassified	Alveolata_unclassified	Alveolata_unclassified
Eukaryota	Opisthokonta	Fungi	Basidiomycota	Agaricomycotina	Agaricomycetes	Agaricomycetes_unclassified	Agaricomycetes_unclassified
Eukaryota	Opisthokonta	Choanoflagellida	Choanoflagellatea	Craspedida	Salpingoecidae_Group_C2	Salpingoecidae_Group_C2_X	Salpingoecidae_Group_C2_X_sp.
Eukaryota	Opisthokonta	Fungi	Chytridiomycota	Chytridiomycotina	Chytridiomycotina_unclassified	Chytridiomycotina_unclassified	Chytridiomycotina_unclassified
Eukaryota	Opisthokonta	Fungi	Cryptomycota	Cryptomycotina	Cryptomycotina_X	Cryptomycotina_XX	Cryptomycotina_XX_sp.
Eukaryota	Stramenopiles	Sagenista	Labyrinthulomycetes	Labyrinthulida	Labyrinthulaceae	Labyrinthulaceae_X	Labyrinthulaceae_X_sp.
Eukaryota	Alveolata	Ciliophora	Spirotrichea	Hypotrichia	Holostichidae	Holosticha	Holosticha_diademata
Eukaryota	Alveolata	Ciliophora	Spirotrichea	Hypotrichia	Pseudourostylidae	Pseudourostyla	Pseudourostyla_cristata
Eukaryota	Alveolata	Ciliophora	Spirotrichea	Hypotrichia	Oxytrichidae	Oxytrichidae_unclassified	Oxytrichidae_unclassified
Eukaryota	Alveolata	Ciliophora	Phyllopharyngea	Cyrtophoria_8	Dysteriidae	Trochilia	Trochilia_petrani
Eukaryota	Alveolata	Ciliophora	Phyllopharyngea	Cyrtophoria_8	Dysteriidae	Trochilia	Trochilia_unclassified
Eukaryota	Opisthokonta	Opisthokonta_unclassified	Opisthokonta_unclassified	Opisthokonta_unclassified	Opisthokonta_unclassified	Opisthokonta_unclassified	Opisthokonta_unclassified
Eukaryota	Alveolata	Ciliophora	Ciliophora_unclassified	Ciliophora_unclassified	Ciliophora_unclassified	Ciliophora_unclassified	Ciliophora_unclassified
Eukaryota	Alveolata	Ciliophora	Oligohymenophorea	Hymenostomatia	Glaucomidae	Glaucomidae_X	Glaucomidae_X_sp.
Eukaryota	Alveolata	Ciliophora	Oligohymenophorea	Hymenostomatia	Glaucomidae	Glaucomidae_unclassified	Glaucomidae_unclassified
Eukaryota	Alveolata	Ciliophora	Oligohymenophorea	Hymenostomatia	Hymenostomatia_unclassified	Hymenostomatia_unclassified	Hymenostomatia_unclassified
Eukaryota	Alveolata	Ciliophora	Oligohymenophorea	Hymenostomatia	Glaucomidae	Glaucoma	Glaucoma_scintillans
Eukaryota	Alveolata	Ciliophora	Oligohymenophorea	OLIGO4	OLIGO4_X	OLIGO4_XX	OLIGO4_XX_sp.
Eukaryota	Alveolata	Ciliophora	Oligohymenophorea	Oligohymenophorea_unclassified	Oligohymenophorea_unclassified	Oligohymenophorea_unclassified	Oligohymenophorea_unclassified
Eukaryota	Alveolata	Ciliophora	Litostomatea	Litostomatea_unclassified	Litostomatea_unclassified	Litostomatea_unclassified	Litostomatea_unclassified
Eukaryota	Alveolata	Ciliophora	Litostomatea	Rhynchostomatia	Tracheliidae	Trachelius	Trachelius_ovum
Eukaryota	Alveolata	Ciliophora	Litostomatea	Haptoria_5	Pleurostomatida	Pleurostomatida_X	Pleurostomatida_X_sp.
Eukaryota	Alveolata	Ciliophora	Litostomatea	Haptoria_5	Pleurostomatida	Pleurostomatida_unclassified	Pleurostomatida_unclassified
Eukaryota	Opisthokonta	Metazoa	Cnidaria	Cnidaria_X	Hydrozoa	Hydra	Hydra_magnipapillata
Eukaryota	Apusozoa	Hilomonadea	Planomonadida	Planomonadidae	Planomonadidae_X	Micronuclearia	Micronuclearia_podoventralis
Eukaryota	Hacrobia	Cryptophyta	Cryptophyceae	Cryptomonadales	Cryptomonadales_X	Cryptomonas	Cryptomonas_unclassified
Eukaryota	Archaeplastida	Archaeplastida_unclassified	Archaeplastida_unclassified	Archaeplastida_unclassified	Archaeplastida_unclassified	Archaeplastida_unclassified	Archaeplastida_unclassified
Eukaryota	Archaeplastida	Chlorophyta	Chlorophyceae	Chlorophyceae_unclassified	Chlorophyceae_unclassified	Chlorophyceae_unclassified	Chlorophyceae_unclassified
Eukaryota	Archaeplastida	Streptophyta	Embryophyceae	Embryophyceae_X	Embryophyceae_XX	Sanionia	Sanionia_uncinata
Eukaryota	Archaeplastida	Streptophyta	Embryophyceae	Embryophyceae_X	Embryophyceae_XX	Embryophyceae_XX_unclassified	Embryophyceae_XX_unclassified
Eukaryota	Archaeplastida	Chlorophyta	Chlorophyceae	Oedogoniales	Oedogoniales_X	Oedogonium	Oedogonium_unclassified
Eukaryota	Archaeplastida	Chlorophyta	Chlorophyceae	Chlamydomonadales	Chlamydomonadales_X	Chlamydomonadales_X_unclassified	Chlamydomonadales_X_unclassified
Eukaryota	Archaeplastida	Chlorophyta	Ulvophyceae	Ulvophyceae_unclassified	Ulvophyceae_unclassified	Ulvophyceae_unclassified	Ulvophyceae_unclassified
Eukaryota	Archaeplastida	Chlorophyta	Trebouxiophyceae	Trebouxiophyceae_unclassified	Trebouxiophyceae_unclassified	Trebouxiophyceae_unclassified	Trebouxiophyceae_unclassified
Eukaryota	Archaeplastida	Chlorophyta	Chlorophyceae	Chlamydomonadales	Chlamydomonadales_X	Chlamydopodium	Chlamydopodium_sp.
Eukaryota	Archaeplastida	Chlorophyta	Trebouxiophyceae	Watanabea-Clade	Watanabea-Clade_X	Chloroidium	Chloroidium_saccharophila
Eukaryota	Archaeplastida	Chlorophyta	Chlorophyceae	Sphaeropleales	Sphaeropleales_X	Sphaeropleales_X_unclassified	Sphaeropleales_X_unclassified
Eukaryota	Archaeplastida	Chlorophyta	Chlorophyceae	Chlamydomonadales	Chlamydomonadales_X	Chlamydomonas	Chlamydomonas_monadina
Eukaryota	Archaeplastida	Chlorophyta	Chlorophyceae	Chlamydomonadales	Chlamydomonadales_X	Chlamydomonas	Chlamydomonas_sordida
Eukaryota	Archaeplastida	Chlorophyta	Chlorophyceae	Chlamydomonadales	Chlamydomonadales_X	Chlamydomonas	Chlamydomonas_unclassified
Eukaryota	Archaeplastida	Chlorophyta	Chlorophyceae	Chaetophorales	Chaetophorales_X	Chaetophora	Chaetophora_incrassata
Eukaryota	Archaeplastida	Chlorophyta	Chlorophyceae	Chaetophorales	Chaetophorales_X	Chaetophorales_X_unclassified	Chaetophorales_X_unclassified
Eukaryota	Archaeplastida	Chlorophyta	Trebouxiophyceae	Trebouxiophyceae_X	Trebouxiophyceae_XX	Trebouxiophyceae_XXX	Trebouxiophyceae_XXX_sp.
Eukaryota	Archaeplastida	Chlorophyta	Ulvophyceae	Ulotrichales	Ulotrichales_X	Ulotrichales_X_unclassified	Ulotrichales_X_unclassified
Eukaryota	Archaeplastida	Chlorophyta	Ulvophyceae	Ulotrichales	Ulotrichales_X	Trichosarcina	Trichosarcina_mucosa
Eukaryota	Archaeplastida	Chlorophyta	Trebouxiophyceae	Prasiolales	Prasiolales_X	Prasiolales_X_unclassified	Prasiolales_X_unclassified
Eukaryota	Archaeplastida	Chlorophyta	Chlorophyta_unclassified	Chlorophyta_unclassified	Chlorophyta_unclassified	Chlorophyta_unclassified	Chlorophyta_unclassified
Eukaryota	Alveolata	Apicomplexa	Gregarinomorphea	Neogregarinorida	Actinocephalidae	Ascogregarina	Ascogregarina_unclassified
Eukaryota	Alveolata	Apicomplexa	Gregarinomorphea	Neogregarinorida	Actinocephalidae	Monocystis	Monocystis_sp.
Eukaryota	Alveolata	Apicomplexa	Gregarinomorphea	Neogregarinorida	Actinocephalidae	Actinocephalidae_unclassified	Actinocephalidae_unclassified
Eukaryota	Alveolata	Apicomplexa	Gregarinomorphea	Neogregarinorida	Actinocephalidae	Syncystis	Syncystis_mirabilis
Eukaryota	Amoebozoa	Lobosa	Tubulinea	Nolandida	Nolandellidae	Nolandellidae_X	Nolandellidae_X_sp.
Eukaryota	Rhizaria	Cercozoa	Filosa-Sarcomonadea	Glissomonadida	Glissomonadida_unclassified	Glissomonadida_unclassified	Glissomonadida_unclassified
Eukaryota	Rhizaria	Cercozoa	Filosa-Sarcomonadea	Glissomonadida	Sandonidae	Sandonidae_unclassified	Sandonidae_unclassified
Eukaryota	Rhizaria	Cercozoa	Filosa-Imbricatea	Filosa-Imbricatea_unclassified	Filosa-Imbricatea_unclassified	Filosa-Imbricatea_unclassified	Filosa-Imbricatea_unclassified
Eukaryota	Rhizaria	Cercozoa	Filosa-Sarcomonadea	Glissomonadida	Glissomonadida_XX	Glissomonadida_XX	Glissomonadida_XX_sp.
Eukaryota	Rhizaria	Cercozoa	Filosa-Sarcomonadea	Filosa-Sarcomonadea_unclassified	Filosa-Sarcomonadea_unclassified	Filosa-Sarcomonadea_unclassified	Filosa-Sarcomonadea_unclassified
Eukaryota	Rhizaria	Cercozoa	Filosa-Imbricatea	Euglyphida	Trinematidae	Trinema	Trinema_enchelys
Eukaryota	Rhizaria	Cercozoa	Filosa-Sarcomonadea	Cercomonadida	Paracercomonadidae	Paracercomonas	Paracercomonas_unclassified
Eukaryota	Rhizaria	Cercozoa	Filosa-Sarcomonadea	Glissomonadida	Sandonidae	Sandona	Sandona_sp.
Eukaryota	Rhizaria	Cercozoa	Filosa-Sarcomonadea	Glissomonadida	Allapsidae	Allapsidae_X	Allapsidae_X_sp.
Eukaryota	Rhizaria	Cercozoa	Cercozoa_unclassified	Cercozoa_unclassified	Cercozoa_unclassified	Cercozoa_unclassified	Cercozoa_unclassified
Eukaryota	Rhizaria	Cercozoa	Filosa-Thecofilosea	Cryomonadida	Rhogostoma-lineage	Rhogostoma-lineage_unclassified	Rhogostoma-lineage_unclassified
Eukaryota	Rhizaria	Cercozoa	Filosa-Thecofilosea	Cryomonadida	Cryomonadida_unclassified	Cryomonadida_unclassified	Cryomonadida_unclassified
Eukaryota	Rhizaria	Cercozoa	Filosa-Thecofilosea	Cryomonadida	Rhogostoma-lineage	Rhogostoma	Rhogostoma_sp.
Eukaryota	Rhizaria	Cercozoa	Filosa-Thecofilosea	Cryomonadida	Rhogostoma-lineage	Rhogostoma-lineage_X	Rhogostoma-lineage_X_sp.

Eukaryota	Alveolata	Ciliophora	Phyllopharyngea	Cyrtophoria_4	Chilodonellidae	Chilodonellidae_unclassified	Chilodonellidae_unclassified
Eukaryota	Alveolata	Ciliophora	Phyllopharyngea	Cyrtophoria_4	Chilodonellidae	Trithigmostoma	Trithigmostoma_steini
Eukaryota	Alveolata	Ciliophora	Phyllopharyngea	Cyrtophoria_4	Chilodonellidae	Chilodonella	Chilodonella_uncinata
Eukaryota	Alveolata	Ciliophora	Phyllopharyngea	Cyrtophoria_2	Lynchellidae_1	Lynchellidae_1_unclassified	Lynchellidae_1_unclassified
Eukaryota	Alveolata	Ciliophora	Phyllopharyngea	Cyrtophoria_2	Lynchellidae_1	Chlamydonella	Chlamydonella_irregularis
Eukaryota	Alveolata	Ciliophora	Oligohymenophorea	Peritrichia_2	Sessilida	Epistylis	Epistylis_plicatilis
Eukaryota	Alveolata	Ciliophora	Oligohymenophorea	Peritrichia_2	Sessilida	Epistylis	Epistylis_unclassified
Eukaryota	Alveolata	Ciliophora	Oligohymenophorea	Peritrichia_2	Sessilida	Vorticella	Vorticella_campanula
Eukaryota	Alveolata	Ciliophora	Oligohymenophorea	Peritrichia_2	Sessilida	Carchesium	Carchesium_polypinum
Eukaryota	Alveolata	Ciliophora	Oligohymenophorea	Peritrichia_2	Sessilida	Sessilida_X	Sessilida_X_sp.
Eukaryota	Alveolata	Ciliophora	Oligohymenophorea	Peritrichia_2	Sessilida	Vorticella	Vorticella_unclassified
Eukaryota	Alveolata	Ciliophora	Oligohymenophorea	Peritrichia_2	Sessilida	Vorticella	Vorticella_aequilata
Eukaryota	Alveolata	Ciliophora	Oligohymenophorea	Peritrichia_2	Sessilida	Sessilida_unclassified	Sessilida_unclassified
Eukaryota	Opisthokonta	Metazoa	Arthropoda	Crustacea	Maxillopoda	Eucyclops	Eucyclops_serrulatus
Eukaryota	Opisthokonta	Metazoa	Arthropoda	Arthropoda_unclassified	Arthropoda_unclassified	Arthropoda_unclassified	Arthropoda_unclassified
Eukaryota	Opisthokonta	Metazoa	Arthropoda	Chelicerata	Arachnida	Arachnida_unclassified	Arachnida_unclassified
Eukaryota	Opisthokonta	Metazoa	Platyhelminthes	Turbellaria	Seriata	Polycelis	Polycelis_nigra
Eukaryota	Opisthokonta	Metazoa	Arthropoda	Hexapoda	Insecta	Insecta_unclassified	Insecta_unclassified
Eukaryota	Opisthokonta	Metazoa	Arthropoda	Hexapoda	Insecta	Hydropsyche	Hydropsyche_sp.
Eukaryota	Opisthokonta	Metazoa	Arthropoda	Hexapoda	Insecta	Baetis	Baetis_harrisoni
Eukaryota	Opisthokonta	Metazoa	Nematoda	Enoplea	Enoplea_X	Enoplea_X_unclassified	Enoplea_X_unclassified
Eukaryota	Opisthokonta	Metazoa	Nematoda	Chromadorea	Chromadorea_X	Chromadorea_X_unclassified	Chromadorea_X_unclassified
Eukaryota	Opisthokonta	Metazoa	Nematoda	Chromadorea	Chromadorea_X	Eumonhystera	Eumonhystera_sp.
Eukaryota	Opisthokonta	Metazoa	Nematoda	Chromadorea	Chromadorea_X	Eumonhystera	Eumonhystera_filiformis
Eukaryota	Opisthokonta	Metazoa	Nematoda	Chromadorea	Chromadorea_X	Monhystera	Monhystera_paludicola
Eukaryota	Opisthokonta	Metazoa	Annelida	Annelida_X	Annelida_XX	Annelida_XX_unclassified	Annelida_XX_unclassified
Eukaryota	Opisthokonta	Metazoa	Rotifera	Rotifera_X	Rotifera_XX	Rotifera_XX_unclassified	Rotifera_XX_unclassified
Eukaryota	Opisthokonta	Metazoa	Arthropoda	Chelicerata	Arachnida	Sperchon	Sperchon_violaceus
Eukaryota	Opisthokonta	Metazoa	Gastrotricha	Gastrotricha_X	Gastrotricha_XX	Chaetonotus	Chaetonotus_aemilianus
Eukaryota	Opisthokonta	Metazoa	Gastrotricha	Gastrotricha_X	Gastrotricha_XX	Heterolepidoderma	Heterolepidoderma_sp.
Eukaryota	Opisthokonta	Metazoa	Gastrotricha	Gastrotricha_X	Gastrotricha_XX	Gastrotricha_XX_unclassified	Gastrotricha_XX_unclassified
Eukaryota	Alveolata	Ciliophora	Spirotrichea	Hypotrichia	Hypotrichia_unclassified	Hypotrichia_unclassified	Hypotrichia_unclassified
Eukaryota	Opisthokonta	Metazoa	Gastrotricha	Gastrotricha_X	Gastrotricha_XX	Heterolepidoderma	Heterolepidoderma_unclassified
Eukaryota	Opisthokonta	Metazoa	Gastrotricha	Gastrotricha_X	Gastrotricha_XX	Lepidodermella	Lepidodermella_minus
Eukaryota	Opisthokonta	Metazoa	Gastrotricha	Gastrotricha_X	Gastrotricha_XX	Chaetonotus	Chaetonotus_acanthodes
Eukaryota	Opisthokonta	Metazoa	Gastrotricha	Gastrotricha_X	Gastrotricha_XX	Chaetonotus	Chaetonotus_unclassified
Eukaryota	Amoebozoa	Lobosa	Tubulinea	Tubulinea_unclassified	Tubulinea_unclassified	Tubulinea_unclassified	Tubulinea_unclassified
Eukaryota	Opisthokonta	Metazoa	Arthropoda	Hexapoda	Insecta	Simulium	Simulium_sanctipauli
Eukaryota	Opisthokonta	Metazoa	Arthropoda	Hexapoda	Insecta	Simulium	Simulium_unclassified
Eukaryota	Opisthokonta	Metazoa	Arthropoda	Hexapoda	Insecta	Atrichopogon	Atrichopogon_minutus
Eukaryota	Opisthokonta	Metazoa	Arthropoda	Hexapoda	Insecta	Eukiefferiella	Eukiefferiella_claripennis
Eukaryota	Opisthokonta	Metazoa	Arthropoda	Hexapoda	Insecta	Orthocladius	Orthocladius_luteipes
Eukaryota	Opisthokonta	Metazoa	Arthropoda	Hexapoda	Insecta	Cricotopus	Cricotopus_sp.
Eukaryota	Opisthokonta	Metazoa	Arthropoda	Hexapoda	Insecta	Pentaneurini	Pentaneurini_sp.
Eukaryota	Opisthokonta	Metazoa	Arthropoda	Hexapoda	Insecta	Paratanytarsus	Paratanytarsus_sp.
Eukaryota	Opisthokonta	Metazoa	Arthropoda	Hexapoda	Insecta	Micropsectra	Micropsectra_sp.
Eukaryota	Opisthokonta	Metazoa	Arthropoda	Hexapoda	Insecta	Corynoneura	Corynoneura_sp.
Eukaryota	Opisthokonta	Metazoa	Arthropoda	Hexapoda	Insecta	Nanocladius	Nanocladius_sp.
Eukaryota	Stramenopiles	Sagenista	Labyrinthulomycetes	Amphifilida	Amphifilaceae	Fibrophrys	Fibrophrys_sp.
Eukaryota	Opisthokonta	Metazoa	Rotifera	Rotifera_X	Rotifera_XX	Rotifera_XXX	Rotifera_XXX_sp.
Eukaryota	Opisthokonta	Metazoa	Bryozoa	Bryozoa_X	Bryozoa_XX	Paludicella	Paludicella_sp.
Eukaryota	Opisthokonta	Metazoa	Metazoa_unclassified	Metazoa_unclassified	Metazoa_unclassified	Metazoa_unclassified	Metazoa_unclassified

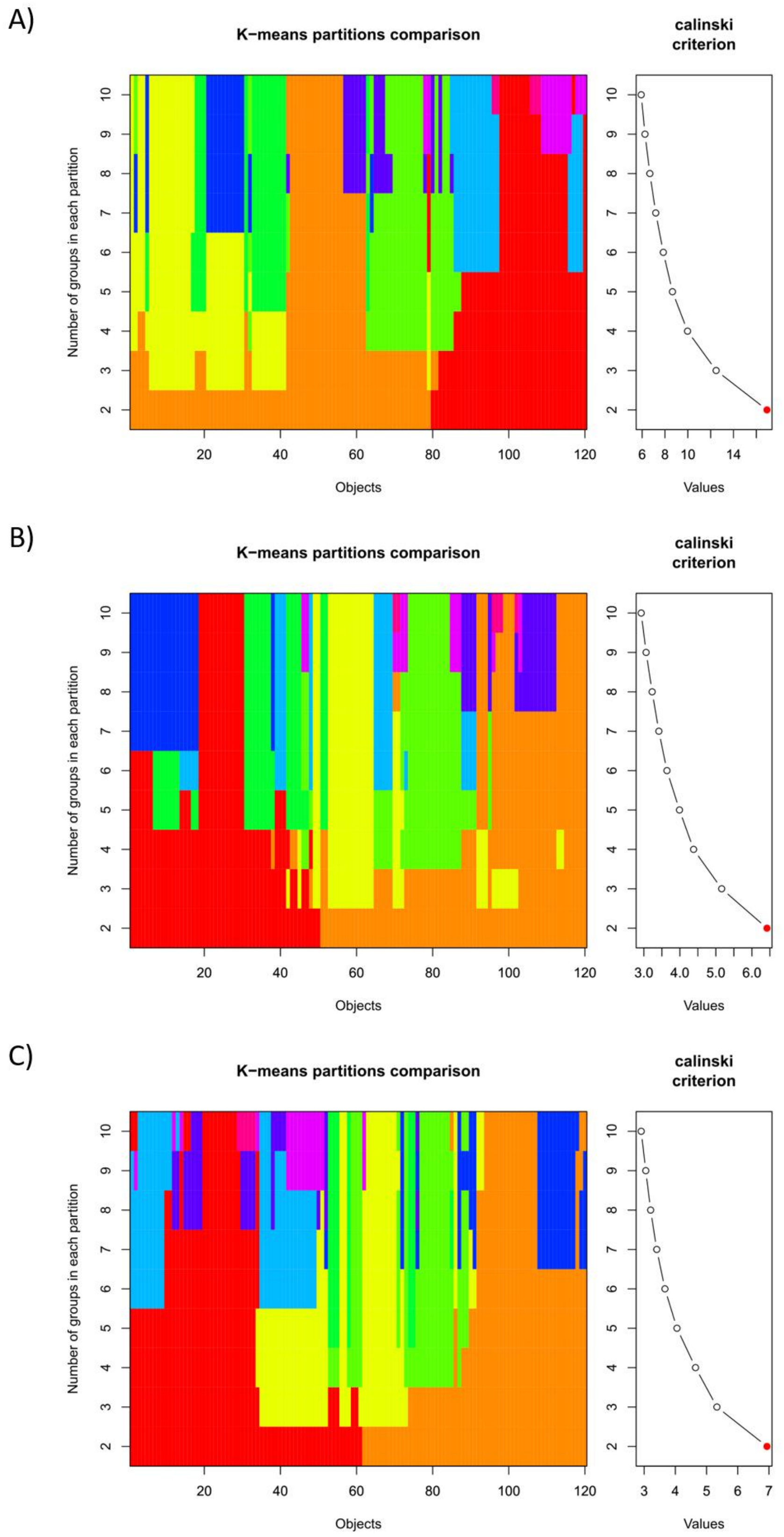

Fig. S1: Results of optimal clustering procedure following k-means partitions of groups and the Kalinski selection criterion for A) bacteria community, B) eukaryote community and C) the joint community including bacteria and eukaryotes. Note that colours among plots are only indicative of the number of groups per partition and do not correspond to similar groups.

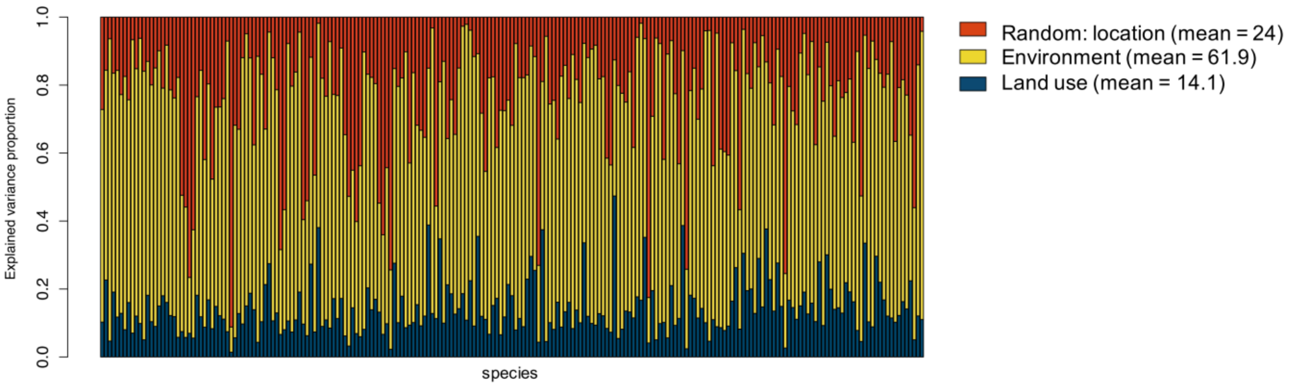

Fig. S2: Results on variance partitioning. Variation in taxon occurrences of m_{joint} is partitioned into responses to fixed and random effects, where all environmental variables i.e., water conductivity, pH, temperature, total phosphorous and N:P are grouped into "Environment". The bar-plot shows taxon-specific results whereas the legend shows averages over all taxa.

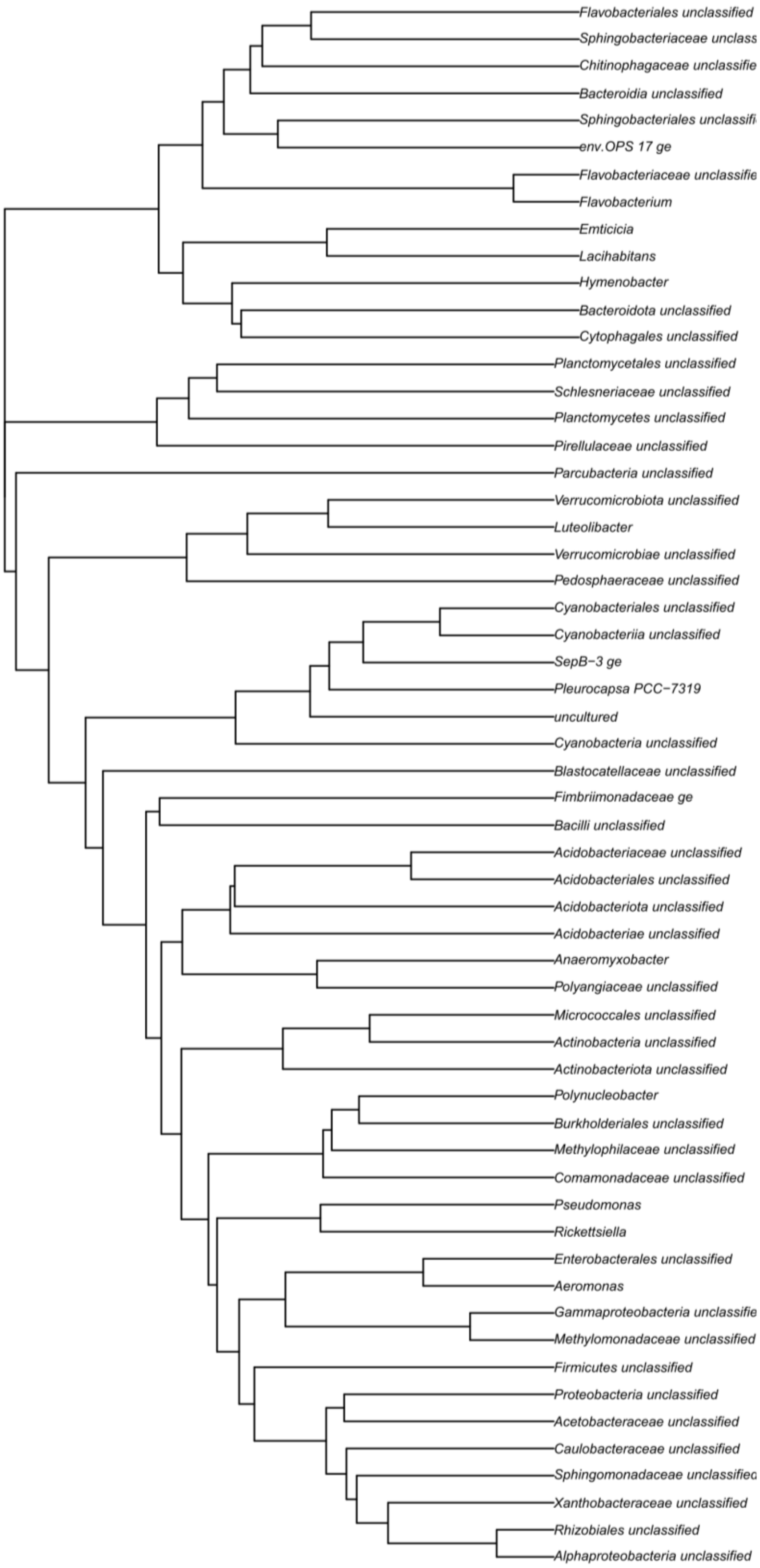

Fig. S3: Phylogenetic tree of included bacteria OTUs

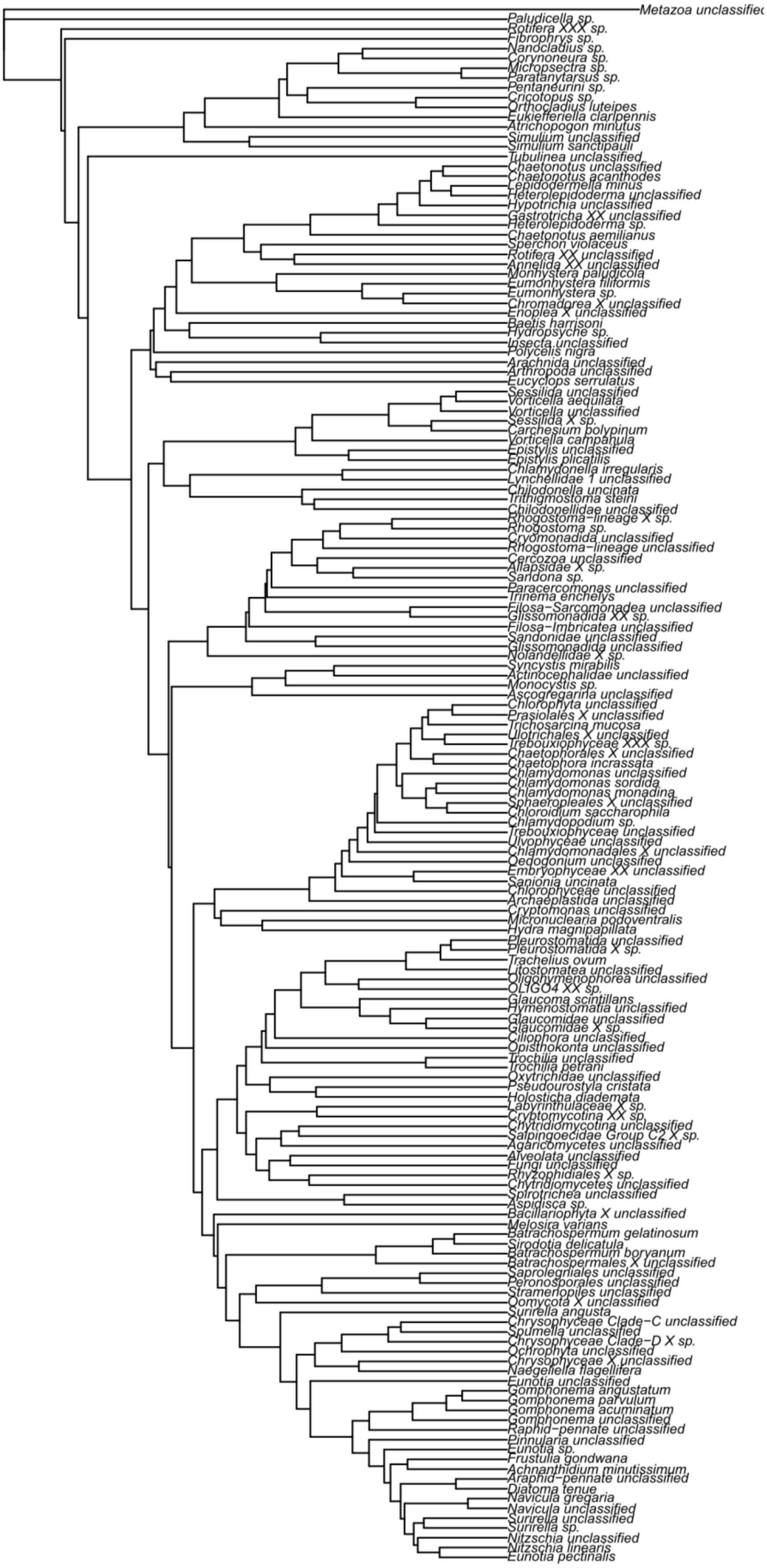

Fig. S4: Phylogenetic tree of included eukaryote OTUs
